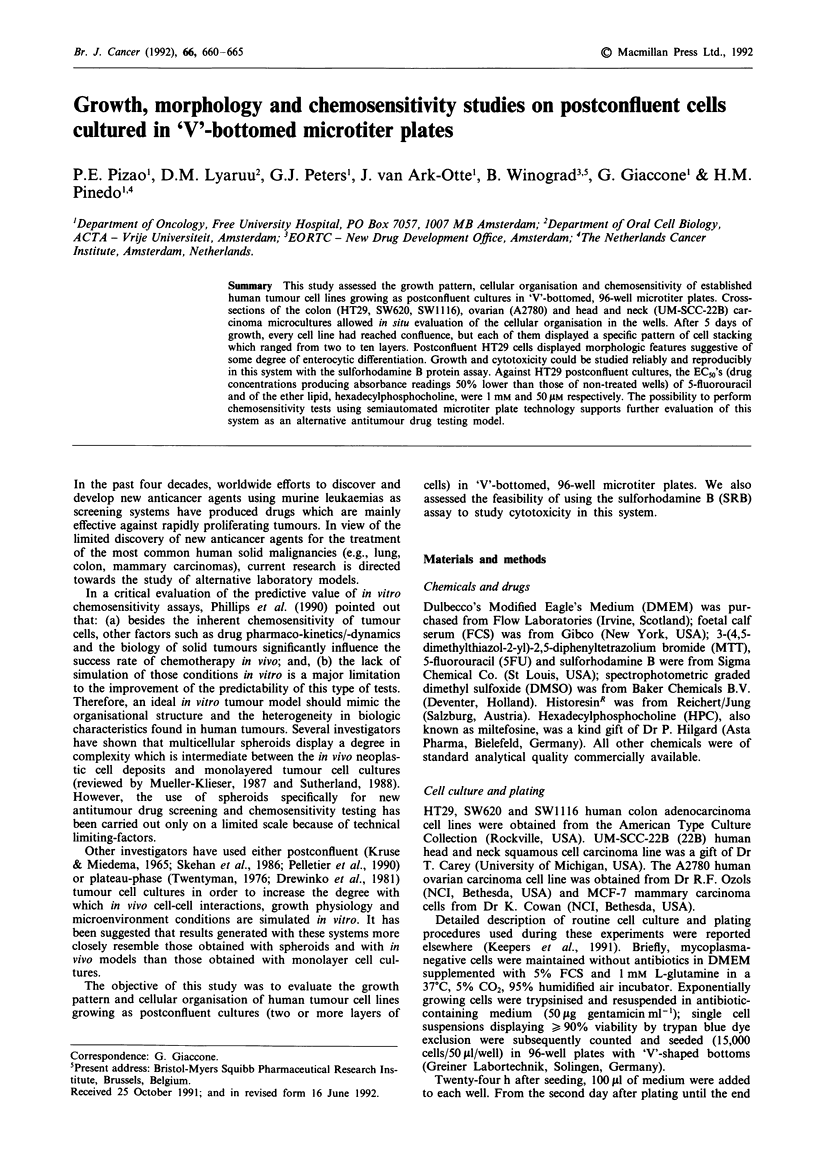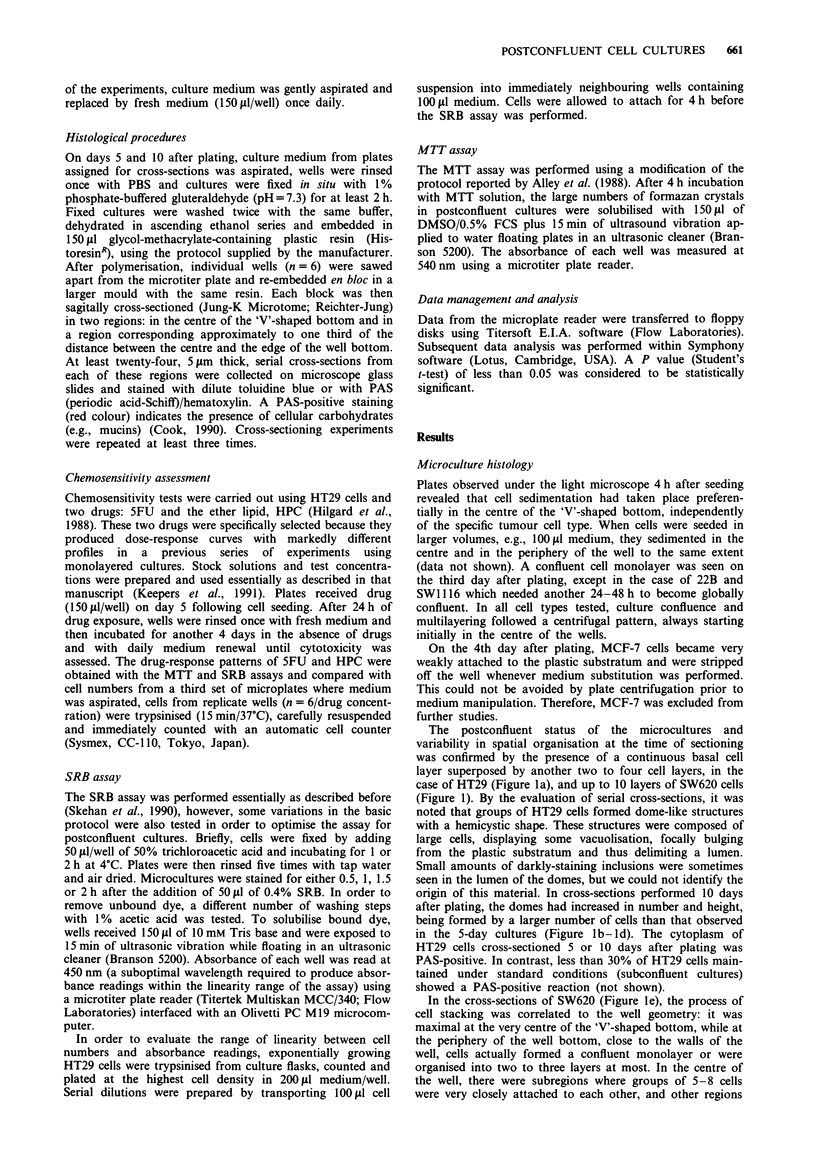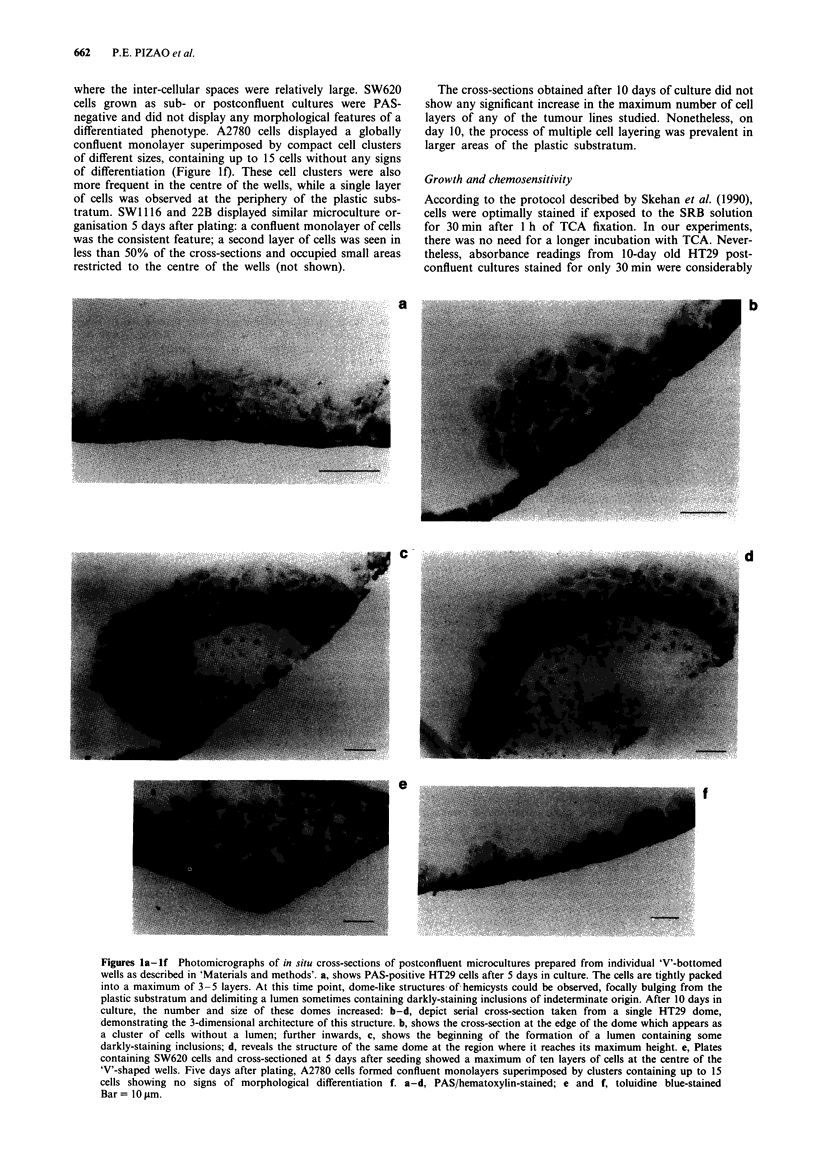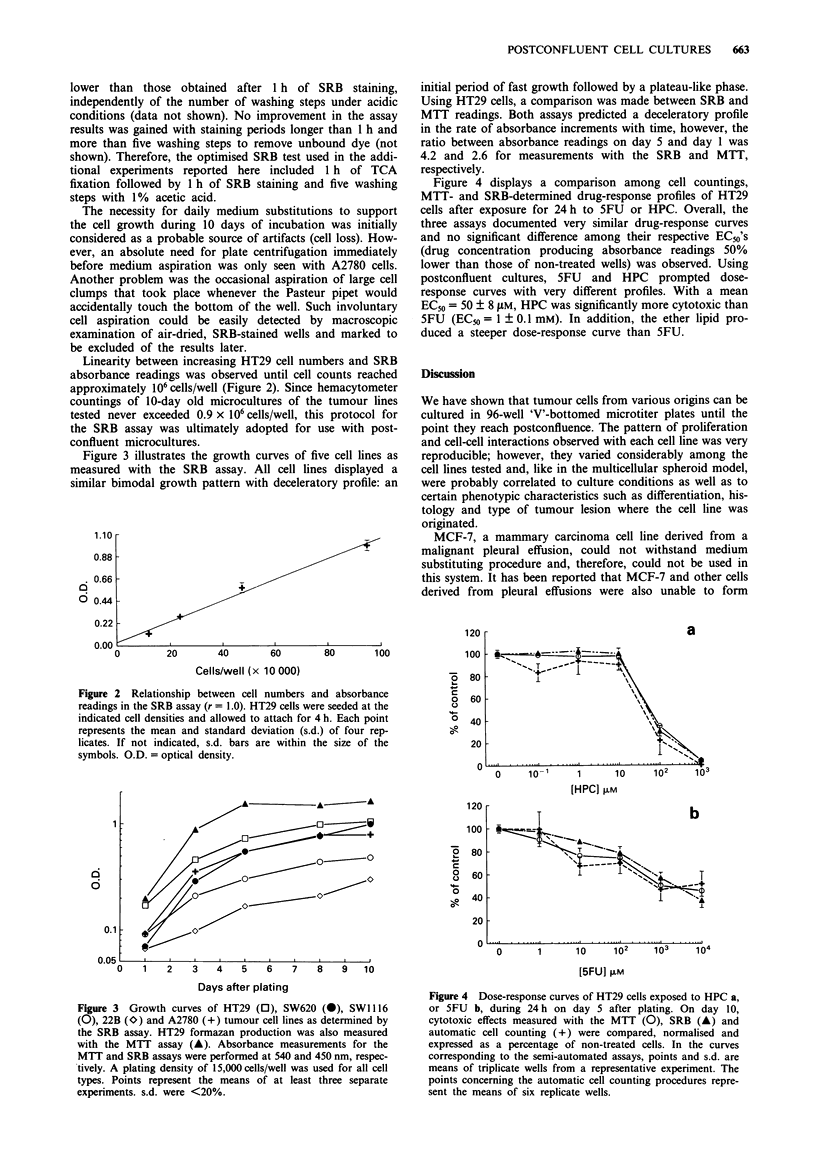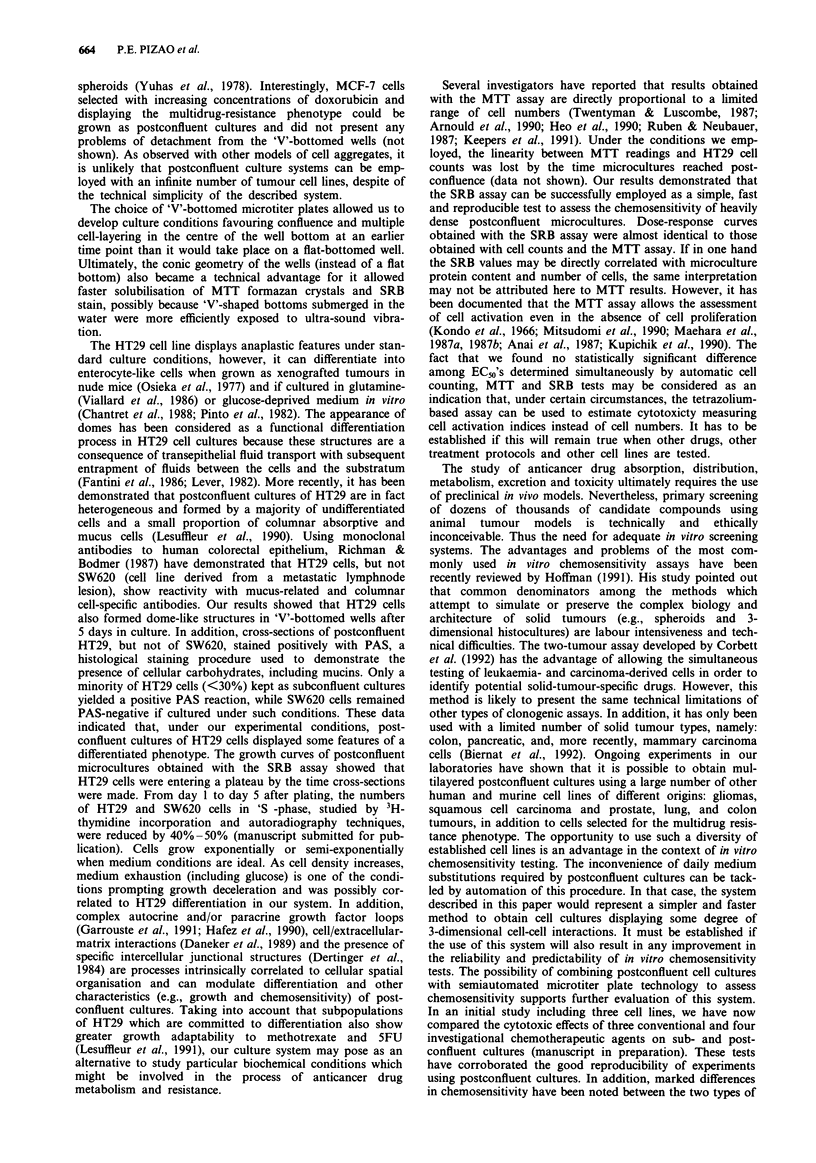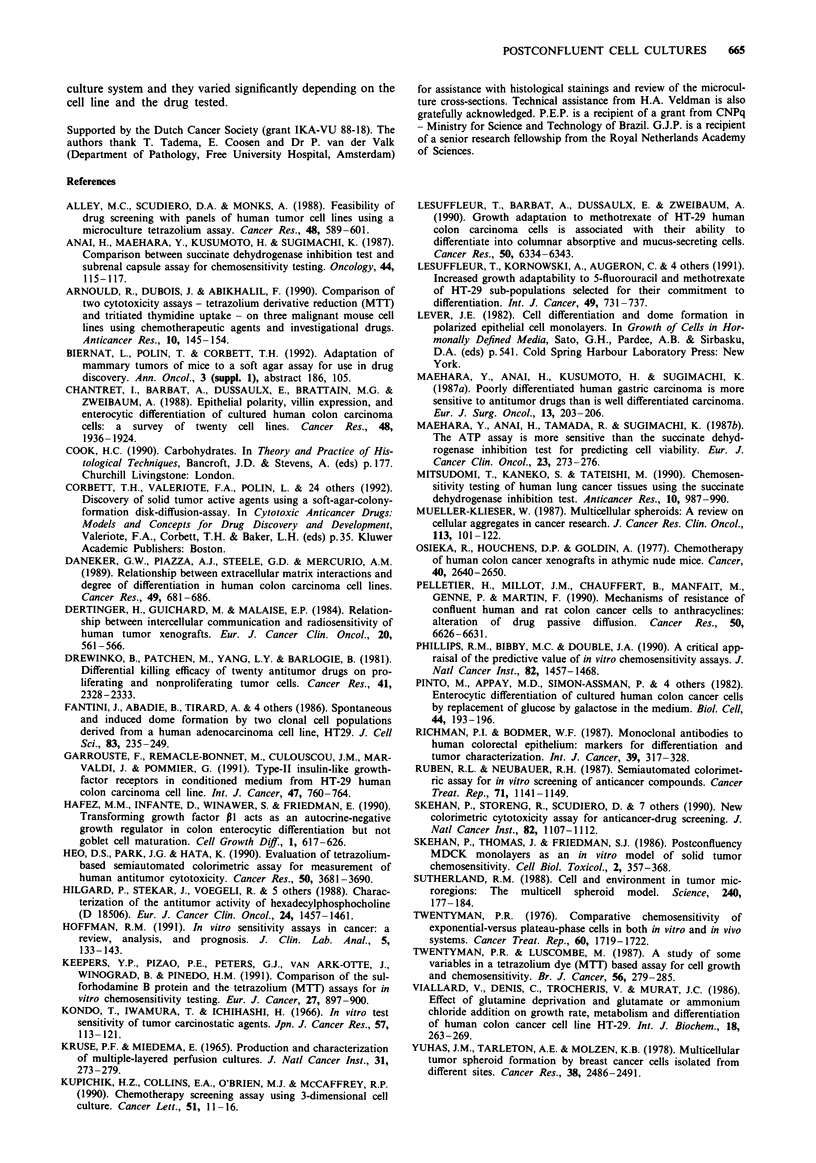# Growth, morphology and chemosensitivity studies on postconfluent cells cultured in 'V'-bottomed microtiter plates.

**DOI:** 10.1038/bjc.1992.333

**Published:** 1992-10

**Authors:** P. E. Pizao, D. M. Lyaruu, G. J. Peters, J. van Ark-Otte, B. Winograd, G. Giaccone, H. M. Pinedo

**Affiliations:** Department of Oncology, Free University Hospital, Amsterdam, Netherlands.

## Abstract

**Images:**


Br. J. Cancer (1992), 66, 660–665 © Macmillan Press Ltd., 1992

### Growth, morphology and chemosensitivity studies on postconfluent cells cultured in 'V'-bottomed microtiter plates

P.E. Pizao[1], D.M. Lyaruu[2], G.J. Peters[1], J. van Ark-Otte[1], B. Winograd[3,5], G. Giaccone[1] & H.M. Pinedo[1,4]

[1]*Department of Oncology, Free University Hospital, PO Box 7057, 1007 MB Amsterdam;* [2]*Department of Oral Cell Biology, ACTA – Vrije Universiteit, Amsterdam;* [3]*EORTC – New Drug Development Office, Amsterdam;* [4]*The Netherlands Cancer Institute, Amsterdam, Netherlands.*

**Summary** This study assessed the growth pattern, cellular organisation and chemosensitivity of established human tumour cell lines growing as postconfluent cultures in 'V'-bottomed, 96-well microtiter plates. Cross-sections of the colon (HT29, SW620, SW1116), ovarian (A2780) and head and neck (UM-SCC-22B) carcinoma microcultures allowed *in situ* evaluation of the cellular organisation in the wells. After 5 days of growth, every cell line had reached confluence, but each of them displayed a specific pattern of cell stacking which ranged from two to ten layers. Postconfluent HT29 cells displayed morphologic features suggestive of some degree of enterocytic differentiation. Growth and cytotoxicity could be studied reliably and reproducibly in this system with the sulforhodamine B protein assay. Against HT29 postconfluent cultures, the $EC_{50}$'s (drug concentrations producing absorbance readings 50% lower than those of non-treated wells) of 5-fluorouracil and of the ether lipid, hexadecylphosphocholine, were 1 mM and 50 µM respectively. The possibility to perform chemosensitivity tests using semiautomated microtiter plate technology supports further evaluation of this system as an alternative antitumour drug testing model.

In the past four decades, worldwide efforts to discover and develop new anticancer agents using murine leukaemias as screening systems have produced drugs which are mainly effective against rapidly proliferating tumours. In view of the limited discovery of new anticancer agents for the treatment of the most common human solid malignancies (e.g., lung, colon, mammary carcinomas), current research is directed towards the study of alternative laboratory models.

In a critical evaluation of the predictive value of *in vitro* chemosensitivity assays, Phillips *et al.* (1990) pointed out that: (a) besides the inherent chemosensitivity of tumour cells, other factors such as drug pharmaco-kinetics/-dynamics and the biology of solid tumours significantly influence the success rate of chemotherapy *in vivo*; and, (b) the lack of simulation of those conditions *in vitro* is a major limitation to the improvement of the predictability of this type of tests. Therefore, an ideal *in vitro* tumour model should mimic the organisational structure and the heterogeneity in biologic characteristics found in human tumours. Several investigators have shown that multicellular spheroids display a degree in complexity which is intermediate between the *in vivo* neoplastic cell deposits and monolayered tumour cell cultures (reviewed by Mueller-Klieser, 1987 and Sutherland, 1988). However, the use of spheroids specifically for new antitumour drug screening and chemosensitivity testing has been carried out only on a limited scale because of technical limiting-factors.

Other investigators have used either postconfluent (Kruse & Miedema, 1965; Skehan *et al.*, 1986; Pelletier *et al.*, 1990) or plateau-phase (Twentyman, 1976; Drewinko *et al.*, 1981) tumour cell cultures in order to increase the degree with which *in vivo* cell-cell interactions, growth physiology and microenvironment conditions are simulated *in vitro*. It has been suggested that results generated with these systems more closely resemble those obtained with spheroids and with *in vivo* models than those obtained with monolayer cell cultures.

The objective of this study was to evaluate the growth pattern and cellular organisation of human tumour cell lines growing as postconfluent cultures (two or more layers of cells) in 'V'-bottomed, 96-well microtiter plates. We also assessed the feasibility of using the sulforhodamine B (SRB) assay to study cytotoxicity in this system.

### Materials and methods

### *Chemicals and drugs*

Dulbecco's Modified Eagle's Medium (DMEM) was purchased from Flow Laboratories (Irvine, Scotland); foetal calf serum (FCS) was from Gibco (New York, USA); 3-(4,5-dimethylthiazol-2-yl)-2,5-diphenyltetrazolium bromide (MTT), 5-fluorouracil (5FU) and sulforhodamine B were from Sigma Chemical Co. (St Louis, USA); spectrophotometric graded dimethyl sulfoxide (DMSO) was from Baker Chemicals B.V. (Deventer, Holland). Historesin[R] was from Reichert/Jung (Salzburg, Austria). Hexadecylphosphocholine (HPC), also known as miltefosine, was a kind gift of Dr P. Hilgard (Asta Pharma, Bielefeld, Germany). All other chemicals were of standard analytical quality commercially available.

### *Cell culture and plating*

HT29, SW620 and SW1116 human colon adenocarcinoma cell lines were obtained from the American Type Culture Collection (Rockville, USA). UM-SCC-22B (22B) human head and neck squamous cell carcinoma line was a gift of Dr T. Carey (University of Michigan, USA). The A2780 human ovarian carcinoma cell line was obtained from Dr R.F. Ozols (NCI, Bethesda, USA) and MCF-7 mammary carcinoma cells from Dr K. Cowan (NCI, Bethesda, USA).

Detailed description of routine cell culture and plating procedures used during these experiments were reported elsewhere (Keepers *et al.*, 1991). Briefly, mycoplasma-negative cells were maintained without antibiotics in DMEM supplemented with 5% FCS and 1 mM L-glutamine in a 37°C, 5% $CO_2$, 95% humidified air incubator. Exponentially growing cells were trypsinised and resuspended in antibiotic-containing medium (50 µg gentamicin $ml^{-1}$); single cell suspensions displaying ≥ 90% viability by trypan blue dye exclusion were subsequently counted and seeded (15,000 cells/50 µl/well) in 96-well plates with 'V'-shaped bottoms (Greiner Labortechnik, Solingen, Germany).

Twenty-four h after seeding, 100 µl of medium were added to each well. From the second day after plating until the end

Correspondence: G. Giaccone.
[5]Present address: Bristol-Myers Squibb Pharmaceutical Research Institute, Brussels, Belgium.
Received 25 October 1991; and in revised form 16 June 1992.

of the experiments, culture medium was gently aspirated and replaced by fresh medium (150 µl/well) once daily.

*Histological procedures*

On days 5 and 10 after plating, culture medium from plates assigned for cross-sections was aspirated, wells were rinsed once with PBS and cultures were fixed *in situ* with 1% phosphate-buffered gluteraldehyde (pH = 7.3) for at least 2 h. Fixed cultures were washed twice with the same buffer, dehydrated in ascending ethanol series and embedded in 150 µl glycol-methacrylate-containing plastic resin (Historesin[R]), using the protocol supplied by the manufacturer. After polymerisation, individual wells ($n = 6$) were sawed apart from the microtiter plate and re-embedded *en bloc* in a larger mould with the same resin. Each block was then sagitally cross-sectioned (Jung-K Microtome; Reichter-Jung) in two regions: in the centre of the 'V'-shaped bottom and in a region corresponding approximately to one third of the distance between the centre and the edge of the well bottom. At least twenty-four, 5 µm thick, serial cross-sections from each of these regions were collected on microscope glass slides and stained with dilute toluidine blue or with PAS (periodic acid-Schiff)/hematoxylin. A PAS-positive staining (red colour) indicates the presence of cellular carbohydrates (e.g., mucins) (Cook, 1990). Cross-sectioning experiments were repeated at least three times.

*Chemosensitivity assessment*

Chemosensitivity tests were carried out using HT29 cells and two drugs: 5FU and the ether lipid, HPC (Hilgard *et al.*, 1988). These two drugs were specifically selected because they produced dose-response curves with markedly different profiles in a previous series of experiments using monolayered cultures. Stock solutions and test concentrations were prepared and used essentially as described in that manuscript (Keepers *et al.*, 1991). Plates received drug (150 µl/well) on day 5 following cell seeding. After 24 h of drug exposure, wells were rinsed once with fresh medium and then incubated for another 4 days in the absence of drugs and with daily medium renewal until cytotoxicity was assessed. The drug-response patterns of 5FU and HPC were obtained with the MTT and SRB assays and compared with cell numbers from a third set of microplates where medium was aspirated, cells from replicate wells ($n = 6$/drug concentration) were trypsinised (15 min/37°C), carefully resuspended and immediately counted with an automatic cell counter (Sysmex, CC-110, Tokyo, Japan).

*SRB assay*

The SRB assay was performed essentially as described before (Skehan *et al.*, 1990), however, some variations in the basic protocol were also tested in order to optimise the assay for postconfluent cultures. Briefly, cells were fixed by adding 50 µl/well of 50% trichloroacetic acid and incubating for 1 or 2 h at 4°C. Plates were then rinsed five times with tap water and air dried. Microcultures were stained for either 0.5, 1, 1.5 or 2 h after the addition of 50 µl of 0.4% SRB. In order to remove unbound dye, a different number of washing steps with 1% acetic acid was tested. To solubilise bound dye, wells received 150 µl of 10 mM Tris base and were exposed to 15 min of ultrasonic vibration while floating in an ultrasonic cleaner (Branson 5200). Absorbance of each well was read at 450 nm (a suboptimal wavelength required to produce absorbance readings within the linearity range of the assay) using a microtiter plate reader (Titertek Multiskan MCC/340; Flow Laboratories) interfaced with an Olivetti PC M19 microcomputer.

In order to evaluate the range of linearity between cell numbers and absorbance readings, exponentially growing HT29 cells were trypsinised from culture flasks, counted and plated at the highest cell density in 200 µl medium/well. Serial dilutions were prepared by transporting 100 µl cell suspension into immediately neighbouring wells containing 100 µl medium. Cells were allowed to attach for 4 h before the SRB assay was performed.

*MTT assay*

The MTT assay was performed using a modification of the protocol reported by Alley *et al.* (1988). After 4 h incubation with MTT solution, the large numbers of formazan crystals in postconfluent cultures were solubilised with 150 µl of DMSO/0.5% FCS plus 15 min of ultrasound vibration applied to water floating plates in an ultrasonic cleaner (Branson 5200). The absorbance of each well was measured at 540 nm using a microtiter plate reader.

*Data management and analysis*

Data from the microplate reader were transferred to floppy disks using Titersoft E.I.A. software (Flow Laboratories). Subsequent data analysis was performed within Symphony software (Lotus, Cambridge, USA). A $P$ value (Student's $t$-test) of less than 0.05 was considered to be statistically significant.

### Results

*Microculture histology*

Plates observed under the light microscope 4 h after seeding revealed that cell sedimentation had taken place preferentially in the centre of the 'V'-shaped bottom, independently of the specific tumour cell type. When cells were seeded in larger volumes, e.g., 100 µl medium, they sedimented in the centre and in the periphery of the well to the same extent (data not shown). A confluent cell monolayer was seen on the third day after plating, except in the case of 22B and SW1116 which needed another 24–48 h to become globally confluent. In all cell types tested, culture confluence and multilayering followed a centrifugal pattern, always starting initially in the centre of the wells.

On the 4th day after plating, MCF-7 cells became very weakly attached to the plastic substratum and were stripped off the well whenever medium substitution was performed. This could not be avoided by plate centrifugation prior to medium manipulation. Therefore, MCF-7 was excluded from further studies.

The postconfluent status of the microcultures and variability in spatial organisation at the time of sectioning was confirmed by the presence of a continuous basal cell layer superposed by another two to four cell layers, in the case of HT29 (Figure 1a), and up to 10 layers of SW620 cells (Figure 1). By the evaluation of serial cross-sections, it was noted that groups of HT29 cells formed dome-like structures with a hemicystic shape. These structures were composed of large cells, displaying some vacuolisation, focally bulging from the plastic substratum and thus delimiting a lumen. Small amounts of darkly-staining inclusions were sometimes seen in the lumen of the domes, but we could not identify the origin of this material. In cross-sections performed 10 days after plating, the domes had increased in number and height, being formed by a larger number of cells than that observed in the 5-day cultures (Figure 1b–1d). The cytoplasm of HT29 cells cross-sectioned 5 or 10 days after plating was PAS-positive. In contrast, less than 30% of HT29 cells maintained under standard conditions (subconfluent cultures) showed a PAS-positive reaction (not shown).

In the cross-sections of SW620 (Figure 1e), the process of cell stacking was correlated to the well geometry: it was maximal at the very centre of the 'V'-shaped bottom, while at the periphery of the well bottom, close to the walls of the well, cells actually formed a confluent monolayer or were organised into two to three layers at most. In the centre of the well, there were subregions where groups of 5–8 cells were very closely attached to each other, and other regions where the inter-cellular spaces were relatively large. SW620 cells grown as sub- or postconfluent cultures were PAS-negative and did not display any morphological features of a differentiated phenotype. A2780 cells displayed a globally confluent monolayer superimposed by compact cell clusters of different sizes, containing up to 15 cells without any signs of differentiation (Figure 1f). These cell clusters were also more frequent in the centre of the wells, while a single layer of cells was observed at the periphery of the plastic substratum. SW1116 and 22B displayed similar microculture organisation 5 days after plating: a confluent monolayer of cells was the consistent feature; a second layer of cells was seen in less than 50% of the cross-sections and occupied small areas restricted to the centre of the wells (not shown).

The cross-sections obtained after 10 days of culture did not show any significant increase in the maximum number of cell layers of any of the tumour lines studied. Nonetheless, on day 10, the process of multiple cell layering was prevalent in larger areas of the plastic substratum.

*Growth and chemosensitivity*

According to the protocol described by Skehan *et al.* (1990), cells were optimally stained if exposed to the SRB solution for 30 min after 1 h of TCA fixation. In our experiments, there was no need for a longer incubation with TCA. Nevertheless, absorbance readings from 10-day old HT29 post-confluent cultures stained for only 30 min were considerably

**Figures 1a–1f** Photomicrographs of *in situ* cross-sections of postconfluent microcultures prepared from individual 'V'-bottomed wells as described in 'Materials and methods'. **a**, shows PAS-positive HT29 cells after 5 days in culture. The cells are tightly packed into a maximum of 3–5 layers. At this time point, dome-like structures of hemicysts could be observed, focally bulging from the plastic substratum and delimiting a lumen sometimes containing darkly-staining inclusions of indeterminate origin. After 10 days in culture, the number and size of these domes increased: **b–d**, depict serial cross-section taken from a single HT29 dome, demonstrating the 3-dimensional architecture of this structure. **b**, shows the cross-section at the edge of the dome which appears as a cluster of cells without a lumen; further inwards, **c**, shows the beginning of the formation of a lumen containing some darkly-staining inclusions; **d**, reveals the structure of the same dome at the region where it reaches its maximum height. **e**, Plates containing SW620 cells and cross-sectioned at 5 days after seeding showed a maximum of ten layers of cells at the centre of the 'V'-shaped wells. Five days after plating, A2780 cells formed confluent monolayers superimposed by clusters containing up to 15 cells showing no signs of morphological differentiation **f**. **a–d**, PAS/hematoxylin-stained; **e** and **f**, toluidine blue-stained Bar = 10 µm.

lower than those obtained after 1 h of SRB staining, independently of the number of washing steps under acidic conditions (data not shown). No improvement in the assay results was gained with staining periods longer than 1 h and more than five washing steps to remove unbound dye (not shown). Therefore, the optimised SRB test used in the additional experiments reported here included 1 h of TCA fixation followed by 1 h of SRB staining and five washing steps with 1% acetic acid.

The necessity for daily medium substitutions to support the cell growth during 10 days of incubation was initially considered as a probable source of artifacts (cell loss). However, an absolute need for plate centrifugation immediately before medium aspiration was only seen with A2780 cells. Another problem was the occasional aspiration of large cell clumps that took place whenever the Pasteur pipet would accidentally touch the bottom of the well. Such involuntary cell aspiration could be easily detected by macroscopic examination of air-dried, SRB-stained wells and marked to be excluded of the results later.

Linearity between increasing HT29 cell numbers and SRB absorbance readings was observed until cell counts reached approximately $10^6$ cells/well (Figure 2). Since hemacytometer countings of 10-day old microcultures of the tumour lines tested never exceeded $0.9 \times 10^6$ cells/well, this protocol for the SRB assay was ultimately adopted for use with postconfluent microcultures.

Figure 3 illustrates the growth curves of five cell lines as measured with the SRB assay. All cell lines displayed a similar bimodal growth pattern with deceleratory profile: an initial period of fast growth followed by a plateau-like phase. Using HT29 cells, a comparison was made between SRB and MTT readings. Both assays predicted a deceleratory profile in the rate of absorbance increments with time, however, the ratio between absorbance readings on day 5 and day 1 was 4.2 and 2.6 for measurements with the SRB and MTT, respectively.

Figure 4 displays a comparison among cell countings, MTT- and SRB-determined drug-response profiles of HT29 cells after exposure for 24 h to 5FU or HPC. Overall, the three assays documented very similar drug-response curves and no significant difference among their respective $EC_{50}$'s (drug concentration producing absorbance readings 50% lower than those of non-treated wells) was observed. Using postconfluent cultures, 5FU and HPC prompted dose-response curves with very different profiles. With a mean $EC_{50} = 50 \pm 8$ μM, HPC was significantly more cytotoxic than 5FU ($EC_{50} = 1 \pm 0.1$ mM). In addition, the ether lipid produced a steeper dose-response curve than 5FU.

**Figure 2** Relationship between cell numbers and absorbance readings in the SRB assay ($r = 1.0$). HT29 cells were seeded at the indicated cell densities and allowed to attach for 4 h. Each point represents the mean and standard deviation (s.d.) of four replicates. If not indicated, s.d. bars are within the size of the symbols. O.D. = optical density.

**Figure 3** Growth curves of HT29 (□), SW620 (●), SW1116 (○), 22B (◇) and A2780 (+) tumour cell lines as determined by the SRB assay. HT29 formazan production was also measured with the MTT assay (▲). Absorbance measurements for the MTT and SRB assays were performed at 540 and 450 nm, respectively. A plating density of 15,000 cells/well was used for all cell types. Points represent the means of at least three separate experiments. s.d. were <20%.

### Discussion

We have shown that tumour cells from various origins can be cultured in 96-well 'V'-bottomed microtiter plates until the point they reach postconfluence. The pattern of proliferation and cell-cell interactions observed with each cell line was very reproducible; however, they varied considerably among the cell lines tested and, like in the multicellular spheroid model, were probably correlated to culture conditions as well as to certain phenotypic characteristics such as differentiation, histology and type of tumour lesion where the cell line was originated.

MCF-7, a mammary carcinoma cell line derived from a malignant pleural effusion, could not withstand medium substituting procedure and, therefore, could not be used in this system. It has been reported that MCF-7 and other cells derived from pleural effusions were also unable to form

**Figure 4** Dose-response curves of HT29 cells exposed to HPC **a**, or 5FU **b**, during 24 h on day 5 after plating. On day 10, cytotoxic effects measured with the MTT (○), SRB (▲) and automatic cell counting (+) were compared, normalised and expressed as a percentage of non-treated cells. In the curves corresponding to the semi-automated assays, points and s.d. are means of triplicate wells from a representative experiment. The points concerning the automatic cell counting procedures represent the means of six replicate wells.

spheroids (Yuhas et al., 1978). Interestingly, MCF-7 cells selected with increasing concentrations of doxorubicin and displaying the multidrug-resistance phenotype could be grown as postconfluent cultures and did not present any problems of detachment from the 'V'-bottomed wells (not shown). As observed with other models of cell aggregates, it is unlikely that postconfluent culture systems can be employed with an infinite number of tumour cell lines, despite of the technical simplicity of the described system.

The choice of 'V'-bottomed microtiter plates allowed us to develop culture conditions favouring confluence and multiple cell-layering in the centre of the well bottom at an earlier time point than it would take place on a flat-bottomed well. Ultimately, the conic geometry of the wells (instead of a flat bottom) also became a technical advantage for it allowed faster solubilisation of MTT formazan crystals and SRB stain, possibly because 'V'-shaped bottoms submerged in the water were more efficiently exposed to ultra-sound vibration.

The HT29 cell line displays anaplastic features under standard culture conditions, however, it can differentiate into enterocyte-like cells when grown as xenografted tumours in nude mice (Osieka et al., 1977) and if cultured in glutamine- (Viallard et al., 1986) or glucose-deprived medium *in vitro* (Chantret et al., 1988; Pinto et al., 1982). The appearance of domes has been considered as a functional differentiation process in HT29 cell cultures because these structures are a consequence of transepithelial fluid transport with subsequent entrapment of fluids between the cells and the substratum (Fantini et al., 1986; Lever, 1982). More recently, it has been demonstrated that postconfluent cultures of HT29 are in fact heterogeneous and formed by a majority of undifferentiated cells and a small proportion of columnar absorptive and mucus cells (Lesuffleur et al., 1990). Using monoclonal antibodies to human colorectal epithelium, Richman & Bodmer (1987) have demonstrated that HT29 cells, but not SW620 (cell line derived from a metastatic lymphnode lesion), show reactivity with mucus-related and columnar cell-specific antibodies. Our results showed that HT29 cells also formed dome-like structures in 'V'-bottomed wells after 5 days in culture. In addition, cross-sections of postconfluent HT29, but not of SW620, stained positively with PAS, a histological staining procedure used to demonstrate the presence of cellular carbohydrates, including mucins. Only a minority of HT29 cells (<30%) kept as subconfluent cultures yielded a positive PAS reaction, while SW620 cells remained PAS-negative if cultured under such conditions. These data indicated that, under our experimental conditions, postconfluent cultures of HT29 cells displayed some features of a differentiated phenotype. The growth curves of postconfluent microcultures obtained with the SRB assay showed that HT29 cells were entering a plateau by the time cross-sections were made. From day 1 to day 5 after plating, the numbers of HT29 and SW620 cells in 'S -phase, studied by $^3$H-thymidine incorporation and autoradiography techniques, were reduced by 40%-50% (manuscript submitted for publication). Cells grow exponentially or semi-exponentially when medium conditions are ideal. As cell density increases, medium exhaustion (including glucose) is one of the conditions prompting growth deceleration and was possibly correlated to HT29 differentiation in our system. In addition, complex autocrine and/or paracrine growth factor loops (Garrouste et al., 1991; Hafez et al., 1990), cell/extracellular-matrix interactions (Daneker et al., 1989) and the presence of specific intercellular junctional structures (Dertinger et al., 1984) are processes intrinsically correlated to cellular spatial organisation and can modulate differentiation and other characteristics (e.g., growth and chemosensitivity) of postconfluent cultures. Taking into account that subpopulations of HT29 which are committed to differentiation also show greater growth adaptability to methotrexate and 5FU (Lesuffleur et al., 1991), our culture system may pose as an alternative to study particular biochemical conditions which might be involved in the process of anticancer drug metabolism and resistance.

Several investigators have reported that results obtained with the MTT assay are directly proportional to a limited range of cell numbers (Twentyman & Luscombe, 1987; Arnould et al., 1990; Heo et al., 1990; Ruben & Neubauer, 1987; Keepers et al., 1991). Under the conditions we employed, the linearity between MTT readings and HT29 cell counts was lost by the time microcultures reached postconfluence (data not shown). Our results demonstrated that the SRB assay can be successfully employed as a simple, fast and reproducible test to assess the chemosensitivity of heavily dense postconfluent microcultures. Dose-response curves obtained with the SRB assay were almost identical to those obtained with cell counts and the MTT assay. If in one hand the SRB values may be directly correlated with microculture protein content and number of cells, the same interpretation may not be attributed here to MTT results. However, it has been documented that the MTT assay allows the assessment of cell activation even in the absence of cell proliferation (Kondo et al., 1966; Mitsudomi et al., 1990; Maehara et al., 1987*a*, 1987*b*; Anai et al., 1987; Kupichik et al., 1990). The fact that we found no statistically significant difference among $EC_{50}$'s determined simultaneously by automatic cell counting, MTT and SRB tests may be considered as an indication that, under certain circumstances, the tetrazolium-based assay can be used to estimate cytotoxicty measuring cell activation indices instead of cell numbers. It has to be established if this will remain true when other drugs, other treatment protocols and other cell lines are tested.

The study of anticancer drug absorption, distribution, metabolism, excretion and toxicity ultimately requires the use of preclinical *in vivo* models. Nevertheless, primary screening of dozens of thousands of candidate compounds using animal tumour models is technically and ethically inconceivable. Thus the need for adequate *in vitro* screening systems. The advantages and problems of the most commonly used *in vitro* chemosensitivity assays have been recently reviewed by Hoffman (1991). His study pointed out that common denominators among the methods which attempt to simulate or preserve the complex biology and architecture of solid tumours (e.g., spheroids and 3-dimensional histocultures) are labour intensiveness and technical difficulties. The two-tumour assay developed by Corbett et al. (1992) has the advantage of allowing the simultaneous testing of leukaemia- and carcinoma-derived cells in order to identify potential solid-tumour-specific drugs. However, this method is likely to present the same technical limitations of other types of clonogenic assays. In addition, it has only been used with a limited number of solid tumour types, namely: colon, pancreatic, and, more recently, mammary carcinoma cells (Biernat et al., 1992). Ongoing experiments in our laboratories have shown that it is possible to obtain multilayered postconfluent cultures using a large number of other human and murine cell lines of different origins: gliomas, squamous cell carcinoma and prostate, lung, and colon tumours, in addition to cells selected for the multidrug resistance phenotype. The opportunity to use such a diversity of established cell lines is an advantage in the context of *in vitro* chemosensitivity testing. The inconvenience of daily medium substitutions required by postconfluent cultures can be tackled by automation of this procedure. In that case, the system described in this paper would represent a simpler and faster method to obtain cell cultures displaying some degree of 3-dimensional cell-cell interactions. It must be established if the use of this system will also result in any improvement in the reliability and predictability of *in vitro* chemosensitivity tests. The possibility of combining postconfluent cell cultures with semiautomated microtiter plate technology to assess chemosensitivity supports further evaluation of this system. In an initial study including three cell lines, we have now compared the cytotoxic effects of three conventional and four investigational chemotherapeutic agents on sub- and post-confluent cultures (manuscript in preparation). These tests have corroborated the good reproducibility of experiments using postconfluent cultures. In addition, marked differences in chemosensitivity have been noted between the two types of culture system and they varied significantly depending on the cell line and the drug tested.

Supported by the Dutch Cancer Society (grant IKA-VU 88-18). The authors thank T. Tadema, E. Coosen and Dr P. van der Valk (Department of Pathology, Free University Hospital, Amsterdam) for assistance with histological stainings and review of the microculture cross-sections. Technical assistance from H.A. Veldman is also gratefully acknowledged. P.E.P. is a recipient of a grant from CNPq – Ministry for Science and Technology of Brazil. G.J.P. is a recipient of a senior research fellowship from the Royal Netherlands Academy of Sciences.

### References

ALLEY, M.C., SCUDIERO, D.A. & MONKS, A. (1988). Feasibility of drug screening with panels of human tumor cell lines using a microculture tetrazolium assay. *Cancer Res.*, **48**, 589–601.

ANAI, H., MAEHARA, Y., KUSUMOTO, H. & SUGIMACHI, K. (1987). Comparison between succinate dehydrogenase inhibition test and subrenal capsule assay for chemosensitivity testing. *Oncology*, **44**, 115–117.

ARNOULD, R., DUBOIS, J. & ABIKHALIL, F. (1990). Comparison of two cytotoxicity assays – tetrazolium derivative reduction (MTT) and tritiated thymidine uptake – on three malignant mouse cell lines using chemotherapeutic agents and investigational drugs. *Anticancer Res.*, **10**, 145–154.

BIERNAT, L., POLIN, T. & CORBETT, T.H. (1992). Adaptation of mammary tumors of mice to a soft agar assay for use in drug discovery. *Ann. Oncol.*, **3 (suppl. 1)**, abstract 186, 105.

CHANTRET, I., BARBAT, A., DUSSAULX, E., BRATTAIN, M.G. & ZWEIBAUM, A. (1988). Epithelial polarity, villin expression, and enterocytic differentiation of cultured human colon carcinoma cells: a survey of twenty cell lines. *Cancer Res.*, **48**, 1936–1924.

COOK, H.C. (1990). Carbohydrates. In *Theory and Practice of Histological Techniques*, Bancroft, J.D. & Stevens, A. (eds) p. 177. Churchill Livingstone: London.

CORBETT, T.H., VALERIOTE, F.A., POLIN, L. & 24 others (1992). Discovery of solid tumor active agents using a soft-agar-colony-formation disk-diffusion-assay. In *Cytotoxic Anticancer Drugs: Models and Concepts for Drug Discovery and Development*, Valeriote, F.A., Corbett, T.H. & Baker, L.H. (eds) p. 35. Kluwer Academic Publishers: Boston.

DANEKER, G.W., PIAZZA, A.J., STEELE, G.D. & MERCURIO, A.M. (1989). Relationship between extracellular matrix interactions and degree of differentiation in human colon carcinoma cell lines. *Cancer Res.*, **49**, 681–686.

DERTINGER, H., GUICHARD, M. & MALAISE, E.P. (1984). Relationship between intercellular communication and radiosensitivity of human tumor xenografts. *Eur. J. Cancer Clin. Oncol.*, **20**, 561–566.

DREWINKO, B., PATCHEN, M., YANG, L.Y. & BARLOGIE, B. (1981). Differential killing efficacy of twenty antitumor drugs on proliferating and nonproliferating tumor cells. *Cancer Res.*, **41**, 2328–2333.

FANTINI, J., ABADIE, B., TIRARD, A. & 4 others (1986). Spontaneous and induced dome formation by two clonal cell populations derived from a human adenocarcinoma cell line, HT29. *J. Cell Sci.*, **83**, 235–249.

GARROUSTE, F., REMACLE-BONNET, M., CULOUSCOU, J.M., MARVALDI, J. & POMMIER, G. (1991). Type-II insulin-like growth-factor receptors in conditioned medium from HT-29 human colon carcinoma cell line. *Int. J. Cancer*, **47**, 760–764.

HAFEZ, M.M., INFANTE, D., WINAWER, S. & FRIEDMAN, E. (1990). Transforming growth factor $\beta 1$ acts as an autocrine-negative growth regulator in colon enterocytic differentiation but not goblet cell maturation. *Cell Growth Diff.*, **1**, 617–626.

HEO, D.S., PARK, J.G. & HATA, K. (1990). Evaluation of tetrazolium-based semiautomated colorimetric assay for measurement of human antitumor cytotoxicity. *Cancer Res.*, **50**, 3681–3690.

HILGARD, P., STEKAR, J., VOEGELI, R. & 5 others (1988). Characterization of the antitumor activity of hexadecylphosphocholine (D 18506). *Eur. J. Cancer Clin. Oncol.*, **24**, 1457–1461.

HOFFMAN, R.M. (1991). *In vitro* sensitivity assays in cancer: a review, analysis, and prognosis. *J. Clin. Lab. Anal.*, **5**, 133–143.

KEEPERS, Y.P., PIZAO, P.E., PETERS, G.J., VAN ARK-OTTE, J., WINOGRAD, B. & PINEDO, H.M. (1991). Comparison of the sulforhodamine B protein and the tetrazolium (MTT) assays for *in vitro* chemosensitivity testing. *Eur. J. Cancer*, **27**, 897–900.

KONDO, T., IWAMURA, T. & ICHIHASHI, H. (1966). *In vitro* test sensitivity of tumor carcinostatic agents. *Jpn. J. Cancer Res.*, **57**, 113–121.

KRUSE, P.F. & MIEDEMA, E. (1965). Production and characterization of multiple-layered perfusion cultures. *J. Natl Cancer Inst.*, **31**, 273–279.

KUPICHIK, H.Z., COLLINS, E.A., O'BRIEN, M.J. & MCCAFFREY, R.P. (1990). Chemotherapy screening assay using 3-dimensional cell culture. *Cancer Lett.*, **51**, 11–16.

LESUFFLEUR, T., BARBAT, A., DUSSAULX, E. & ZWEIBAUM, A. (1990). Growth adaptation to methotrexate of HT-29 human colon carcinoma cells is associated with their ability to differentiate into columnar absorptive and mucus-secreting cells. *Cancer Res.*, **50**, 6334–6343.

LESUFFLEUR, T., KORNOWSKI, A., AUGERON, C. & 4 others (1991). Increased growth adaptability to 5-fluorouracil and methotrexate of HT-29 sub-populations selected for their commitment to differentiation. *Int. J. Cancer*, **49**, 731–737.

LEVER, J.E. (1982). Cell differentiation and dome formation in polarized epithelial cell monolayers. In *Growth of Cells in Hormonally Defined Media*, Sato, G.H., Pardee, A.B. & Sirbasku, D.A. (eds) p. 541. Cold Spring Harbour Laboratory Press: New York.

MAEHARA, Y., ANAI, H., KUSUMOTO, H. & SUGIMACHI, K. (1987*a*). Poorly differentiated human gastric carcinoma is more sensitive to antitumor drugs than is well differentiated carcinoma. *Eur. J. Surg. Oncol.*, **13**, 203–206.

MAEHARA, Y., ANAI, H., TAMADA, R. & SUGIMACHI, K. (1987*b*). The ATP assay is more sensitive than the succinate dehydrogenase inhibition test for predicting cell viability. *Eur. J. Cancer Clin. Oncol.*, **23**, 273–276.

MITSUDOMI, T., KANEKO, S. & TATEISHI, M. (1990). Chemosensitivity testing of human lung cancer tissues using the succinate dehydrogenase inhibition test. *Anticancer Res.*, **10**, 987–990.

MUELLER-KLIESER, W. (1987). Multicellular spheroids: A review on cellular aggregates in cancer research. *J. Cancer Res. Clin. Oncol.*, **113**, 101–122.

OSIEKA, R., HOUCHENS, D.P. & GOLDIN, A. (1977). Chemotherapy of human colon cancer xenografts in athymic nude mice. *Cancer*, **40**, 2640–2650.

PELLETIER, H., MILLOT, J.M., CHAUFFERT, B., MANFAIT, M., GENNE, P. & MARTIN, F. (1990). Mechanisms of resistance of confluent human and rat colon cancer cells to anthracyclines: alteration of drug passive diffusion. *Cancer Res.*, **50**, 6626–6631.

PHILLIPS, R.M., BIBBY, M.C. & DOUBLE, J.A. (1990). A critical appraisal of the predictive value of *in vitro* chemosensitivity assays. *J. Natl Cancer Inst.*, **82**, 1457–1468.

PINTO, M., APPAY, M.D., SIMON-ASSMAN, P. & 4 others (1982). Enterocytic differentiation of cultured human colon cancer cells by replacement of glucose by galactose in the medium. *Biol. Cell*, **44**, 193–196.

RICHMAN, P.I. & BODMER, W.F. (1987). Monoclonal antibodies to human colorectal epithelium: markers for differentiation and tumor characterization. *Int. J. Cancer*, **39**, 317–328.

RUBEN, R.L. & NEUBAUER, R.H. (1987). Semiautomated colorimetric assay for *in vitro* screening of anticancer compounds. *Cancer Treat. Rep.*, **71**, 1141–1149.

SKEHAN, P., STORENG, R., SCUDIERO, D. & 7 others (1990). New colorimetric cytotoxicity assay for anticancer-drug screening. *J. Natl Cancer Inst.*, **82**, 1107–1112.

SKEHAN, P., THOMAS, J. & FRIEDMAN, S.J. (1986). Postconfluency MDCK monolayers as an *in vitro* model of solid tumor chemosensitivity. *Cell Biol. Toxicol.*, **2**, 357–368.

SUTHERLAND, R.M. (1988). Cell and environment in tumor microregions: The multicell spheroid model. *Science*, **240**, 177–184.

TWENTYMAN, P.R. (1976). Comparative chemosensitivity of exponential-versus plateau-phase cells in both *in vitro* and *in vivo* systems. *Cancer Treat. Rep.*, **60**, 1719–1722.

TWENTYMAN, P.R. & LUSCOMBE, M. (1987). A study of some variables in a tetrazolium dye (MTT) based assay for cell growth and chemosensitivity. *Br. J. Cancer*, **56**, 279–285.

VIALLARD, V., DENIS, C., TROCHERIS, V. & MURAT, J.C. (1986). Effect of glutamine deprivation and glutamate or ammonium chloride addition on growth rate, metabolism and differentiation of human colon cancer cell line HT-29. *Int. J. Biochem.*, **18**, 263–269.

YUHAS, J.M., TARLETON, A.E. & MOLZEN, K.B. (1978). Multicellular tumor spheroid formation by breast cancer cells isolated from different sites. *Cancer Res.*, **38**, 2486–2491.